# FungiExp: a user-friendly database and analysis platform for exploring fungal gene expression and alternative splicing

**DOI:** 10.1093/bioinformatics/btad042

**Published:** 2023-01-19

**Authors:** Jinding Liu, Yaru Zhang, Yapin Shi, Yiqing Zheng, Yali Zhu, Zhuoran Guan, Danyu Shen, Daolong Dou

**Affiliations:** Bioinformatics Center, Academy for Advanced Interdisciplinary Studies, Nanjing Agricultural University, Nanjing, Jiangsu 210095, China; Department of Animal Science, Michigan State University, East Lansing, MI 48824, USA; College of Information Management, Nanjing Agricultural University, Nanjing, Jiangsu 210095, China; College of Information Management, Nanjing Agricultural University, Nanjing, Jiangsu 210095, China; College of Information Management, Nanjing Agricultural University, Nanjing, Jiangsu 210095, China; College of Information Management, Nanjing Agricultural University, Nanjing, Jiangsu 210095, China; College of Information Management, Nanjing Agricultural University, Nanjing, Jiangsu 210095, China; Department of Plant Pathology, Nanjing Agricultural University, Nanjing, Jiangsu 210095, China; Department of Plant Pathology, Nanjing Agricultural University, Nanjing, Jiangsu 210095, China

## Abstract

**Summary:**

Fungi form a large and heterogeneous group of eukaryotic organisms with diverse ecological niches. The high importance of fungi contrasts with our limited understanding of fungal lifestyle and adaptability to environment. Over the last decade, the high-throughput sequencing technology produced tremendous RNA-sequencing (RNA-seq) data. However, there is no comprehensive database for mycologists to conveniently explore fungal gene expression and alternative splicing. Here, we have developed FungiExp, an online database including 35 821 curated RNA-seq samples derived from 220 fungal species, together with gene expression and alternative splicing profiles. It allows users to query and visualize gene expression and alternative splicing in the collected RNA-seq samples. Furthermore, FungiExp contains several online analysis tools, such as differential/specific, co-expression network and cross-species gene expression conservation analysis. Through these tools, users can obtain new insights by re-analyzing public RNA-seq data or upload personal data to co-analyze with public RNA-seq data.

**Availability and implementation:**

The FungiExp is freely available at https://bioinfo.njau.edu.cn/fungiExp.

**Supplementary information:**

Supplementary data are available at *Bioinformatics* online.

## 1 Introduction

Fungi are a diverse kingdom with 2.2–3.8 million species on Earth (Hawksworth and Lucking, 2017). With the diverse functions, more and more fungi have been discovered and investigated (Valverde *et al.*, 2015). RNA sequencing (RNA-seq) is increasingly used to investigate regulation mechanisms of gene expression and alternative splicing in fungi. Over the last decade, a large number of fungal raw RNA-seq datasets have been produced and deposited in some public databases, for example, the Sequence Read Archive (SRA) database (Katz *et al.*, 2022). However, no comprehensive fungal transcriptomic database simultaneously covers gene expression and alternative splicing profiles.

Here, we present FungiExp, a fungal gene expression and alternative splicing database. It comprises up to 35 821 RNA-seq samples from 220 fungal species whose genomic data were extracted from EnsemblFungi. It has several important features. First, it includes both gene expression and alternative splicing. Second, it is equipped with multiple online analysis modules including differential expression, specific expression, co-expression network and cross-species expression conservation analyses. Third, it allows users to perform re-analysis of public RNA-seq datasets or co-analysis with one's personal datasets. Fourth, a wide range of data visualization tools help users intuitively understand retrieval and analysis results.

## 2 Data collection and database construction

The fungal species deposited in the EnsemblFungi database (Release 47) were candidate species for construction of the FungiExp database. Only RNA-seq data generated from Illumina platforms were considered because of the ubiquity and high base-calling accuracy. The RNA-seq raw datasets were retrieved by querying the SRA database using terms Platform="Illumina", Strategy="RNA-seq" and Source="Transcriptomic". The retrieved RNA-seq datasets were gathered with further manual curation of sample information. Finally, only 220 fungal species with at least three RNA-seq samples available were collected in the FungiExp database (Supplementary Table S1).

StringTie v2.1.4 was used to estimate gene expression levels at two metrics transcripts per million and fragments per kilo base per million mapped reads. The program rMATs v4.0.2 was used to detect five types of alternative splicing events, including alternative 5′ splice site, alternative 3′ splice site, intron retention, skipping exon, and mutually exclusive exon, and percentage spliced in was used to estimate alternative splicing profiles (Shen *et al.*, 2014).

Two popular methods, DESeq2 (Love *et al.*, 2014) and edgeR (Robinson *et al.*, 2010), were integrated to detect differentially expressed genes. SVA was used to adjust the batch effects from different studies (Leek *et al.*, 2012). WGCNA was used for weighted gene co-expression network analysis (Langfelder and Horvath, 2008). To explore gene expression conservation, the ortholog gene expression levels were normalized as the log2-transformed ratios of the expression of a gene in a sample divided by the trimmed mean expression level. Hypergeometry test and gene set enrichment analysis were used to summarize the function distribution of genes of interest (Subramanian *et al.*, 2005; Yu *et al.*, 2015).

## 3 User interfaces

FungiExp is hosted at https://bionfo.njau.edu.cn/fungiExp. It is equipped with various querying (Search and Blast) and analysis (Comparison, Specificity, Coexpression and Cross-species) pages to facilitate the access of the database.

In the search page, users can query genes to display expression and alternative splicing profiles by gene identifier, symbol or functional annotation terms (Supplementary Fig. S1a). Alternatively, in the blast page, genes can be queried by sequence similarity (Supplementary Fig. S1b). The retrieved genes are displayed in a concise table with links to open gene page showing expression and alternative splicing profiles.

In the opened gene page, gene functional annotation terms are firstly listed at the top of the page with links for exploration of the details in external databases (Supplementary Fig. S1c). The following section is the genome browser JBrowse for users to explore genes and alternative splicing events in genomic context (Supplementary Fig. S1d). Next, a structure graph displays functional domains in the translated protein and impacts of alternative splicing on transcript (Supplementary Fig. S1e). Finally, a listed box containing the collected RNA-seq studies and a hierarchical bar chart visually showing gene expression or alternative splicing profiles in samples or sample groups, locate at the bottom of the gene page (Supplementary Fig. S1f).

In the comparison and specificity pages, users can flexibly customize sample groups of interest, analysis tools and parameters to explore differentially/specifically expressed or spliced genes. Users can also upload personal RNA-seq datasets pre-prepared according to our online manual, and perform co-analysis with public RNA-seq datasets. In the returned result page, there are heatmap (Supplementary Fig. S2a) and principal component analysis graph (Supplementary Fig. S2b) to show sample clustering based on global gene expression or alternative splicing profiles. The differentially/specifically expressed and spliced genes are listed in interactive tables with links to open pages showing its details (Supplementary Fig. S2c). The enriched Gene Ontology (GO) terms and Kyoto Encyclopedia of Genes and Genomes (KEGG) pathways on differentially/specifically expressed/spliced genes are shown in two bar graphs (Supplementary Fig. S2d and S2e). For specific expression analysis, a column chart is used to summarize specifically high/low-expressed or spliced genes (Supplementary Fig. S2f).

In the co-expression page, users can customize multiple sample groups to find gene clusters (also module or network) with highly correlated expression profiles. The genes in a cluster tend to be associated with specific sample phenotype or trait. FungiExp provides some graphs to visually show analysis results, such as a heatmap showing relationships between gene clusters and sample groups (Supplementary Fig. S3a), a gene dendrogram of clustered dissimilarity and module color (Supplementary Fig. S3b), network graphs exhibiting gene clusters in which the larger nodes represent the more important hub genes (Supplementary Fig. S3c).

In the cross-species page, users can customize sample groups with similar attributes in any two specified species to explore gene expression conservation. FungiExp offers an interactive table to list ortholog groups with the correlation coefficients representing the consistency of ortholog gene expression intra-species and inter-species (Supplementary Fig. S3d). For an ortholog gene group of interest, users can further open a new page to build a molecular phylogenetic tree and compare gene expression profiles across customized samples (Supplementary Fig. S3e). In addition, FungiExp also provides scatter plots of gene expression ratios of 1:1 ortholog gene pairs in all two-group comparisons (Supplementary Fig. S3f).

## 4 A case study to explore gene expression and alternative splicing during *Ustilaginoidea virens* infection

To illustrate the practicability of FungiExp, we present a case study based on the public RNA-seq dataset (SRP188527) to explore specifically expressed and spliced genes during the infection of *U.virens*, which causes rice false smut (Tang *et al.*, 2021). This study contained four sample groups with two replicates across four infection stages including 1-day post-infection (dpi), 3 dpi, 6 dpi and 15 dpi, respectively. The analysis results returned by FungiExp showed that the eight samples were well clustered into four groups corresponding to the four infection stages based on overall gene expression (Supplementary Fig. S4a) and alternative splicing profiles (Supplementary Fig. S4b), suggesting both gene expression and alternative splicing were affected in the infection period.

With default methods and parameters, FungiExp reported a total of 1483 stage-specifically high-/low-expressed genes and 125 stage-specifically alternative splicing events. The stage-specifically expressed genes and alternative splicing events were gradually increased with infection time (Supplementary Fig. S4c and d). Enrichment analysis results showed the stage-specifically expressed genes (Supplementary Tables S2 and S3) and the stage-specifically spliced genes (Supplementary Tables S4 and S5) were significantly related to fungal infection. The above analysis results, not reported by the original study (Tang *et al.*, 2021), could provide clues for the subsequent pathogenesis studies of *U.virens*.

## 5 Conclusion

FungiExp is a freely available fungal transcriptomic database and analysis platform. Users can obtain new insights by re-analyzing public RNA-seq data or upload personal data to co-analyze with public RNA-seq data. In the future, we plan to add more publicly available fungal RNA-seq data to maintain FungiExp as an up-to-date resource.

## Supplementary Material

btad042_Supplementary_Data
