# Supplementary material for: FungiExp: a user-friendly database and analysis platform for exploring fungal gene expression and alternative splicing
Source: Bioinformatics. 2023 Jan 19;39(1):btad042. doi: 10.1093/bioinformatics/btad042 (PMC9887077; doi:10.1093/bioinformatics/btad042)
Supplement: btad042_Supplementary_Data [file btad042_supplementary_data.doc]

# FungiExp: a user-friendly database and analysis platform for exploring fungal gene expression and alternative splicing

Jinding Liu, Yaru Zhang, Yapin Shi, Yiqing Zheng, Yali Zhu, Zhuoran Guan,

Danyu Shen, Daolong Dou

**Supplementary Tables**

**Supplementary Table S1. Summary of fungal species with RNA-seq datasets**

| **class** | **Species** | **Mapped bases (GB)** | **Study** | **Experiment** |
| --- | --- | --- | --- | --- |
| Acidomyces | *Acidomyces richmondensis* | 44.11 | 1 | 12 |
| Agaricales | *Agaricus bisporus* | 185.27 | 4 | 63 |
|  | *Amanita muscaria* | 61.57 | 8 | 12 |
|  | *Armillaria gallica* | 27.66 | 1 | 6 |
|  | *Armillaria ostoyae* | 344.91 | 2 | 41 |
|  | *Coprinopsis cinerea* | 951.71 | 61 | 232 |
|  | *Grifola frondosa* | 0.97 | 1 | 18 |
|  | *Hebeloma cylindrosporum* | 134.00 | 4 | 28 |
|  | *Hypsizygus marmoreus* | 56.56 | 4 | 11 |
|  | *Laccaria bicolor* | 259.33 | 64 | 80 |
|  | *Lentinula edodes* | 665.17 | 19 | 163 |
|  | *Pleurotus ostreatus* | 391.85 | 10 | 111 |
|  | *Schizophyllum commune* | 179.80 | 6 | 79 |
|  | *Piloderma croceum* | 99.87 | 8 | 27 |
| Blastocladiales | *Allomyces macrogynus* | 4.59 | 1 | 6 |
| Boletales | *Coniophora puteana* | 11.96 | 1 | 6 |
|  | *Hydnomerulius pinastri* | 9.37 | 1 | 6 |
|  | *Paxillus involutus* | 64.21 | 8 | 27 |
|  | *Pisolithus microcarpus* | 174.42 | 4 | 40 |
|  | *Pisolithus tinctorius* | 47.91 | 7 | 7 |
| Botryosphaeriales | *Macrophomina phaseolina* | 73.70 | 3 | 13 |
|  | *Neofusicoccum parvum* | 31.51 | 1 | 9 |
| Cantharellales | *Rhizoctonia solani* | 173.26 | 15 | 98 |
|  | *Tulasnella calospora* | 96.86 | 3 | 13 |
| Capnodiales | *Cercospora zeina* | 16.89 | 1 | 7 |
|  | *Mycosphaerella eumusae* | 5.05 | 1 | 4 |
|  | *Pseudocercospora fijiensis* | 11.07 | 1 | 6 |
|  | *Pseudocercospora musae* | 4.36 | 1 | 4 |
|  | *Ramularia collo-cygni* | 32.68 | 2 | 315 |
|  | *Zymoseptoria tritici* | 405.86 | 31 | 129 |
| Chaetothyriomycetidae | *Exophiala dermatitidis* | 269.38 | 4 | 52 |
|  | *Exophiala spinifera* | 46.60 | 2 | 13 |
| Corticiales | *Punctularia strigosozonata* | 84.60 | 1 | 9 |
| Cystofilobasidiales | *Xanthophyllomyces dendrorhous* | 286.63 | 41 | 63 |
| Dothideales | *Aureobasidium pullulans* | 10.82 | 3 | 8 |
| Erysiphales | *Blumeria graminis* | 51.40 | 9 | 126 |
| Eurotiales | *Aspergillus calidoustus* | 36.89 | 1 | 9 |
|  | *Aspergillus carbonarius* | 80.34 | 4 | 44 |
|  | *Aspergillus cristatus* | 82.41 | 2 | 15 |
|  | *Aspergillus flavus* | 647.95 | 29 | 209 |
|  | *Aspergillus fumigatus* | 2617.60 | 69 | 963 |
|  | *Aspergillus glaucus* | 11.18 | 2 | 15 |
|  | *Aspergillus lacticoffeatus* | 3919.13 | 881 | 1270 |
|  | *Aspergillus luchuensis* | 81.29 | 5 | 30 |
|  | *Aspergillus nidulans* | 977.18 | 94 | 361 |
|  | *Aspergillus niger* | 3770.21 | 861 | 1251 |
|  | *Aspergillus oryzae* | 266.73 | 10 | 94 |
|  | *Aspergillus sydowii* | 129.97 | 5 | 24 |
|  | *Aspergillus terreus* | 28.23 | 4 | 15 |
|  | *Aspergillus tubingensis* | 42.23 | 1 | 5 |
|  | *Aspergillus wentii* | 26.82 | 1 | 3 |
|  | *Penicillium brasilianum* | 26.17 | 1 | 6 |
|  | *Penicillium chrysogenum* | 47.79 | 3 | 16 |
|  | *Penicillium coprophilum* | 22.04 | 1 | 6 |
|  | *Penicillium decumbens* | 23.12 | 2 | 8 |
|  | *Penicillium digitatum* | 170.21 | 9 | 32 |
|  | *Penicillium expansum* | 189.44 | 19 | 47 |
|  | *Penicillium flavigenum* | 26.45 | 1 | 6 |
|  | *Penicillium nalgiovense* | 21.21 | 1 | 6 |
|  | *Penicillium oxalicum* | 397.53 | 14 | 155 |
|  | *Penicillium polonicum* | 20.31 | 1 | 6 |
|  | *Penicillium roqueforti* | 29.03 | 1 | 6 |
|  | *Penicillium steckii* | 26.16 | 1 | 6 |
|  | *Penicillium subrubescens* | 130.22 | 24 | 24 |
| Gloeophyllales | *Gloeophyllum trabeum* | 95.52 | 3 | 22 |
| Glomerales | *Rhizophagus clarus* | 53.07 | 2 | 12 |
|  | *Rhizophagus irregularis* | 242.66 | 14 | 148 |
| Glomerellales | *Colletotrichum gloeosporioides* | 131.50 | 8 | 43 |
|  | *Colletotrichum graminicola* | 20.52 | 12 | 46 |
|  | *Colletotrichum higginsianum* | 38.39 | 12 | 59 |
|  | *Colletotrichum incanum* | 14.04 | 1 | 3 |
|  | *Colletotrichum nymphaeae* | 7.15 | 9 | 9 |
|  | *Verticillium dahliae* | 209.82 | 17 | 118 |
|  | *Verticillium longisporum* | 12.96 | 2 | 17 |
|  | *Colletotrichum tofieldiae* | 0.00 | 1 | 3 |
| Helotiales | *Diplocarpon rosae* | 0.00 | 1 | 3 |
|  | *Glarea lozoyensis* | 31.18 | 2 | 6 |
|  | *Meliniomyces bicolor* | 66.12 | 1 | 6 |
|  | *Pezoloma ericae* | 91.44 | 2 | 7 |
|  | *Phialocephala subalpina* | 20.65 | 1 | 30 |
| Hymenochaetales | *Phellinus noxius* | 51.77 | 2 | 18 |
| Hypocreales | *Acremonium chrysogenum* | 56.41 | 3 | 16 |
|  | *Beauveria bassiana* | 345.45 | 26 | 154 |
|  | *Cordyceps militaris* | 191.87 | 10 | 37 |
|  | *Fusarium culmorum* | 55.02 | 1 | 6 |
|  | *Fusarium graminearum* | 1683.29 | 82 | 508 |
|  | *Fusarium mangiferae* | 1.44 | 1 | 4 |
|  | *Fusarium oxysporum* | 863.34 | 44 | 247 |
|  | *Fusarium poae* | 47.94 | 1 | 7 |
|  | *Fusarium proliferatum* | 33.22 | 3 | 11 |
|  | *Fusarium pseudograminearum* | 73.32 | 3 | 39 |
|  | *Fusarium solani* | 94.43 | 5 | 22 |
|  | *Fusarium verticillioides* | 618.71 | 12 | 195 |
|  | *Metarhizium acridum* | 207.73 | 6 | 47 |
|  | *Metarhizium anisopliae* | 39.37 | 4 | 14 |
|  | *Metarhizium robertsii* | 167.76 | 9 | 55 |
|  | *Ophiocordyceps sinensis* | 420.93 | 12 | 69 |
|  | *Ophiocordyceps unilateralis* | 9.52 | 1 | 3 |
|  | *Pochonia chlamydosporia* | 48.77 | 2 | 11 |
|  | *Purpureocillium lilacinum* | 153.18 | 3 | 17 |
|  | *Trichoderma asperellum* | 60.55 | 3 | 14 |
|  | *Trichoderma atroviride* | 240.30 | 10 | 91 |
|  | *Trichoderma citrinoviride* | 38.04 | 2 | 9 |
|  | *Trichoderma guizhouense* | 89.37 | 2 | 21 |
|  | *Trichoderma harzianum* | 179.13 | 11 | 56 |
|  | *Trichoderma reesei* | 1053.38 | 130 | 331 |
|  | *Ustilaginoidea virens* | 120.14 | 6 | 32 |
| Jaapiales | *Jaapia argillacea* | 14.50 | 1 | 6 |
| Magnaporthales | *Gaeumannomyces tritici* | 42.65 | 4 | 21 |
|  | *Magnaporthe oryzae* | 838.30 | 49 | 518 |
|  | *Magnaporthe poae* | 42.07 | 1 | 14 |
| Malasseziales | *Malassezia globosa* | 48.69 | 2 | 28 |
|  | *Malassezia sympodialis* | 108.32 | 3 | 16 |
| Microsporidia | *Edhazardia aedis* | 20.34 | 11 | 33 |
|  | *Nematocida parisii* | 20.94 | 1 | 23 |
| Mortierellomycotina | *Mortierella elongata* | 221.61 | 11 | 22 |
| Mucoromycotina | *Lichtheimia corymbifera* | 58.56 | 2 | 13 |
|  | *Mucor circinelloides* | 6.09 | 9 | 20 |
|  | *Rhizopus delemar* | 346.26 | 7 | 85 |
|  | *Rhizopus microsporus* | 101.85 | 5 | 57 |
| Myxotrichaceae | *Oidiodendron maius* | 59.25 | 3 | 14 |
| Neocallimastigales | *Anaeromyces robustus* | 368.69 | 83 | 126 |
|  | *Neocallimastix californiae* | 271.94 | 80 | 117 |
|  | *Piromyces finnis* | 59.69 | 2 | 39 |
| Nosematidae | *Nosema bombycis* | 50.77 | 3 | 39 |
|  | *Nosema ceranae* | 52.14 | 2 | 26 |
| Onygenales | *Blastomyces dermatitidis* | 54.11 | 1 | 9 |
|  | *Coccidioides posadasii* | 14.83 | 3 | 16 |
|  | *Histoplasma capsulatum* | 52.31 | 4 | 111 |
|  | *Paracoccidioides brasiliensis* | 8.35 | 2 | 11 |
|  | *Trichophyton benhamiae* | 11.66 | 1 | 15 |
|  | *Trichophyton mentagrophytes* | 45.98 | 1 | 9 |
|  | *Trichophyton rubrum* | 109.98 | 11 | 47 |
| Ophiostomatales | *Grosmannia clavigera* | 66.37 | 2 | 12 |
| Orbiliales | *Arthrobotrys oligospora* | 47.84 | 2 | 14 |
|  | *Drechslerella stenobrocha* | 4.20 | 1 | 3 |
| Pleistophoridae | *Trachipleistophora hominis* | 0.00 | 1 | 13 |
|  | *Vavraia culicis* | 10.62 | 6 | 15 |
| Pleosporales | *Alternaria alternata* | 452.62 | 13 | 111 |
|  | *Ascochyta rabiei* | 101.50 | 1 | 20 |
|  | *Bipolaris maydis* | 142.08 | 3 | 52 |
|  | *Bipolaris sorokiniana* | 104.48 | 21 | 33 |
|  | *Leptosphaeria maculans* | 112.46 | 6 | 73 |
|  | *Phaeosphaeria nodorum* | 43.62 | 1 | 8 |
|  | *Pyrenophora teres* | 34.08 | 2 | 23 |
| Polyporales | *Daedalea quercina* | 14.01 | 8 | 8 |
|  | *Dichomitus squalens* | 756.41 | 280 | 322 |
|  | *Fibroporia radiculosa* | 16.32 | 16 | 22 |
|  | *Ganoderma sinense* | 9.63 | 1 | 3 |
|  | *Phanerochaete carnosa* | 14.88 | 1 | 20 |
|  | *Phlebiopsis gigantea* | 101.45 | 27 | 34 |
|  | *Postia placenta* | 401.54 | 14 | 355 |
|  | *Trametes coccinea* | 222.06 | 4 | 59 |
|  | *Trametes versicolor* | 172.05 | 5 | 55 |
|  | *Wolfiporia cocos* | 70.34 | 12 | 28 |
|  | *Yarrowia lipolytica* | 1139.48 | 142 | 339 |
| Pseudeurotiaceae | *Pseudogymnoascus destructans* | 159.63 | 5 | 35 |
| Pucciniales | *Melampsora larici-populina* | 72.54 | 2 | 15 |
|  | *Puccinia coronata* | 128.51 | 1 | 30 |
|  | *Puccinia graminis* | 87.38 | 5 | 101 |
|  | *Puccinia striiformis* | 1031.18 | 13 | 489 |
|  | *Puccinia triticina* | 104.85 | 4 | 25 |
| Rhizophydiales | *Batrachochytrium dendrobatidis* | 70.13 | 4 | 40 |
| Russulales | *Heterobasidion irregulare* | 5.95 | 1 | 19 |
| Saccharomycetales | *Brettanomyces bruxellensis* | 74.86 | 5 | 34 |
|  | *Candida albicans* | 3291.09 | 171 | 2248 |
|  | *Candida auris* | 362.14 | 9 | 101 |
|  | *Candida dubliniensis* | 11.06 | 2 | 7 |
|  | *Candida glabrata* | 490.56 | 25 | 174 |
|  | *Candida intermedia* | 115.24 | 1 | 27 |
|  | *Candida orthopsilosis* | 25.93 | 3 | 12 |
|  | *Candida parapsilosis* | 321.04 | 13 | 155 |
|  | *Candida tropicalis* | 265.02 | 15 | 73 |
|  | *Clavispora lusitaniae* | 85.14 | 3 | 25 |
|  | *Cyberlindnera jadinii* | 10.41 | 1 | 5 |
|  | *Debaryomyces hansenii* | 0.72 | 3 | 14 |
|  | *Kluyveromyces lactis* | 40.43 | 8 | 67 |
|  | *Kluyveromyces marxianus* | 276.16 | 9 | 86 |
|  | *Komagataella pastoris* | 250.16 | 6 | 139 |
|  | *Komagataella phaffii* | 512.02 | 13 | 396 |
|  | *Lachancea thermotolerans* | 12.25 | 2 | 6 |
|  | *Lipomyces starkeyi* | 99.56 | 3 | 17 |
|  | *Naumovozyma castellii* | 62.17 | 8 | 57 |
|  | *Ogataea parapolymorpha* | 146.73 | 3 | 22 |
|  | *Ogataea polymorpha* | 75.84 | 3 | 68 |
|  | *Pichia kudriavzevii* | 441.12 | 78 | 90 |
|  | *Saccharomyces cerevisiae* | 24091.85 | 915 | 17353 |
|  | *Schizosaccharomyces pombe* | 4010.09 | 163 | 2993 |
|  | *Torulaspora delbrueckii* | 42.33 | 2 | 33 |
|  | *Wickerhamomyces anomalus* | 104.32 | 3 | 36 |
|  | *Zygosaccharomyces bailii* | 14.51 | 2 | 21 |
|  | *Zygosaccharomyces parabailii* | 94.37 | 2 | 18 |
| Schizosaccharomycetales | *Schizosaccharomyces japonicus* | 32.26 | 10 | 14 |
| Sclerotiniaceae | *Botrytis cinerea* | 793.73 | 23 | 231 |
|  | *Sclerotinia sclerotiorum* | 427.89 | 25 | 232 |
| Sebacinales | *Serendipita vermifera* | 23.22 | 3 | 10 |
|  | *Chaetomium globosum* | 50.81 | 4 | 31 |
|  | *Chaetomium thermophilum* | 16.52 | 1 | 3 |
|  | *Neurospora crassa* | 12244.32 | 1918 | 3001 |
|  | *Neurospora tetrasperma* | 175.79 | 3 | 45 |
|  | *Podospora anserina* | 149.33 | 25 | 55 |
|  | *Sordaria macrospora* | 43.59 | 3 | 21 |
|  | *Thermothelomyces thermophila* | 8.60 | 1 | 15 |
| Sordariomycetidae | *Coniochaeta ligniaria* | 159.97 | 2 | 22 |
|  | *Phaeoacremonium minimum* | 57.03 | 3 | 37 |
|  | *Valsa mali* | 46.49 | 3 | 13 |
| Sporidiobolales | *Rhodotorula toruloides* | 373.05 | 86 | 164 |
| Taphrinomycotina | *Pneumocystis carinii* | 4.89 | 2 | 4 |
|  | *Pneumocystis murina* | 1.92 | 2 | 5 |
| Tremellales | *Cryptococcus gattii* | 0.46 | 2 | 9 |
|  | *Cryptococcus neoformans* | 1242.84 | 78 | 1437 |
| Trichosporonales | *Cutaneotrichosporon oleaginosum* | 111.02 | 2 | 15 |
|  | *Trichosporon asahii* | 51.80 | 3 | 16 |
| Tubulinosematidae | *Anncaliia algerae* | 2.86 | 12 | 26 |
| Unikaryonidae | *Encephalitozoon cuniculi* | 3.76 | 1 | 3 |
| Ustilaginales | *Kalmanozyma brasiliensis* | 47.86 | 1 | 10 |
|  | *Moesziomyces aphidis* | 107.72 | 2 | 18 |
|  | *Ustilago maydis* | 210.48 | 10 | 60 |
| Xylariales | *Rosellinia necatrix* | 76.64 | 2 | 12 |
| **Sum** |  | **67896.61** | **5025** | **35821** |

**Supplementary Table S2. GO terms enriched on stage-specifically expressed genes of *U. virens***

| **Stage** | **Class** | **GO term** | **Description** | **pvalue** | **p.adjust** | **qvalue** |
| --- | --- | --- | --- | --- | --- | --- |
| 1 dpi | BP | GO:0006865 | amino acid transport | 0.003981 | 0.0322 | 0.0279 |
| 1 dpi | BP | GO:0009405 | pathogenesis | 0.001901 | 0.0184 | 0.016 |
| 1 dpi | BP | GO:0006032 | chitin catabolic process | 0.001407 | 0.0152 | 0.0132 |
| 1 dpi | BP | GO:0008152 | metabolic process | 0.000735 | 0.0119 | 0.0103 |
| 1 dpi | BP | GO:0006334 | nucleosome assembly | 1.18E-05 | 0.0003 | 0.0002 |
| 1 dpi | BP | GO:0055085 | transmembrane transport | 4.18E-09 | 4.06E-07 | 3.52E-07 |
| 1 dpi | MF | GO:0008236 | serine-type peptidase activity | 0.003539 | 0.0312 | 0.0271 |
| 1 dpi | MF | GO:0004568 | chitinase activity | 0.001407 | 0.0152 | 0.0132 |
| 1 dpi | MF | GO:0031177 | phosphopantetheine binding | 0.001407 | 0.0152 | 0.0132 |
| 1 dpi | MF | GO:0004252 | serine-type endopeptidase activity | 0.000313 | 0.0061 | 0.0053 |
| 1 dpi | MF | GO:0022857 | transmembrane transporter activity | 4.70E-08 | 2.28E-06 | 1.98E-06 |
| 1 dpi | CC | GO:0005576 | extracellular region | 0.004617 | 0.0345 | 0.0299 |
| 1 dpi | CC | GO:0000786 | nucleosome | 1.18E-05 | 0.0003 | 0.0002 |
| 6 dpi | BP | GO:0006412 | translation | 0.000467 | 0.0128 | 0.0113 |
| 6 dpi | BP | GO:0005975 | carbohydrate metabolic process | 0.000209 | 0.008 | 0.007 |
| 6 dpi | BP | GO:0071555 | cell wall organization | 1.46E-05 | 0.0028 | 0.0025 |
| 6 dpi | MF | GO:0004553 | hydrolase activity, hydrolyzing O-glycosyl compounds | 0.00255 | 0.0487 | 0.043 |
| 6 dpi | MF | GO:0005506 | iron ion binding | 0.002122 | 0.045 | 0.0397 |
| 6 dpi | MF | GO:0020037 | heme binding | 0.000742 | 0.0177 | 0.0156 |
| 6 dpi | MF | GO:0003735 | structural constituent of ribosome | 7.17E-05 | 0.0046 | 0.004 |
| 6 dpi | CC | GO:0005576 | extracellular region | 0.000468 | 0.0128 | 0.0113 |
| 6 dpi | CC | GO:0005840 | ribosome | 0.000148 | 0.007 | 0.0062 |
| 6 dpi | CC | GO:0005618 | cell wall | 3.57E-05 | 0.0034 | 0.003 |
| 15 dpi | BP | GO:0055085 | transmembrane transport | 0.000425 | 0.0454 | 0.0431 |
| 15 dpi | MF | GO:0016491 | oxidoreductase activity | 2.76E-05 | 0.0059 | 0.0056 |

**Supplementary Table S3. KEGG pathways enriched on stage-specifically**

**expressed genes of *U. virens***

| **Stage** | **ID** | **Description** | **pvalue** | **p.adjust** | **qvalue** |
| --- | --- | --- | --- | --- | --- |
| stage2 | ko00280 | Valine, leucine and isoleucine degradation | 0.000109397 | 0.004594687 | 0.003800117 |
| stage3 | ko00900 | Terpenoid backbone biosynthesis | 0.002408229 | 0.036725494 | 0.03232097 |
| stage3 | ko01110 | Biosynthesis of secondary metabolites | 0.001367806 | 0.027812057 | 0.02447653 |
| stage3 | ko00100 | Steroid biosynthesis | 0.000288475 | 0.008798479 | 0.007743269 |
| stage3 | ko03010 | Ribosome | 1.62E-09 | 9.88E-08 | 8.70E-08 |
| stage4 | ko00500 | Starch and sucrose metabolism | 1.01E-05 | 0.00069803 | 0.000649578 |

**Supplementary Table S4. GO terms enriched on stage-specifically spliced genes of *U. virens***

| **Stage** | **Class** | **GO term** | **Description** | **pvalue** | **p.adjust** | **qvalue** |
| --- | --- | --- | --- | --- | --- | --- |
| 1 dpi | BP | GO:0018215 | protein phosphopantetheinylation | 0.02092708 | 0.04185416 | 0.011014253 |
| 1 dpi | BP | GO:0000160 | phosphorelay signal transduction system | 0.006462671 | 0.025850685 | 0.006802812 |
| 1 dpi | CC | GO:0005622 | intracellular | 0.036477167 | 0.048636222 | 0.012799006 |

**Supplementary Table S5. KEGG pathways enriched on stage-specifically**

**spliced genes of *U. virens***

| **Stage** | **ID** | **Description** | **pvalue** | **p.adjust** | **qvalue** |
| --- | --- | --- | --- | --- | --- |
| 1 dpi | ko00460 | Cyanoamino acid metabolism | 0.011431745 | 0.034196656 | 0.011998827 |
| 1 dpi | ko00480 | Glutathione metabolism | 0.022797771 | 0.034196656 | 0.011998827 |
| 6 dpi | ko03015 | mRNA surveillance pathway | 0.002986937 | 0.014934683 | 0.012576575 |

**Supplementary Figures**


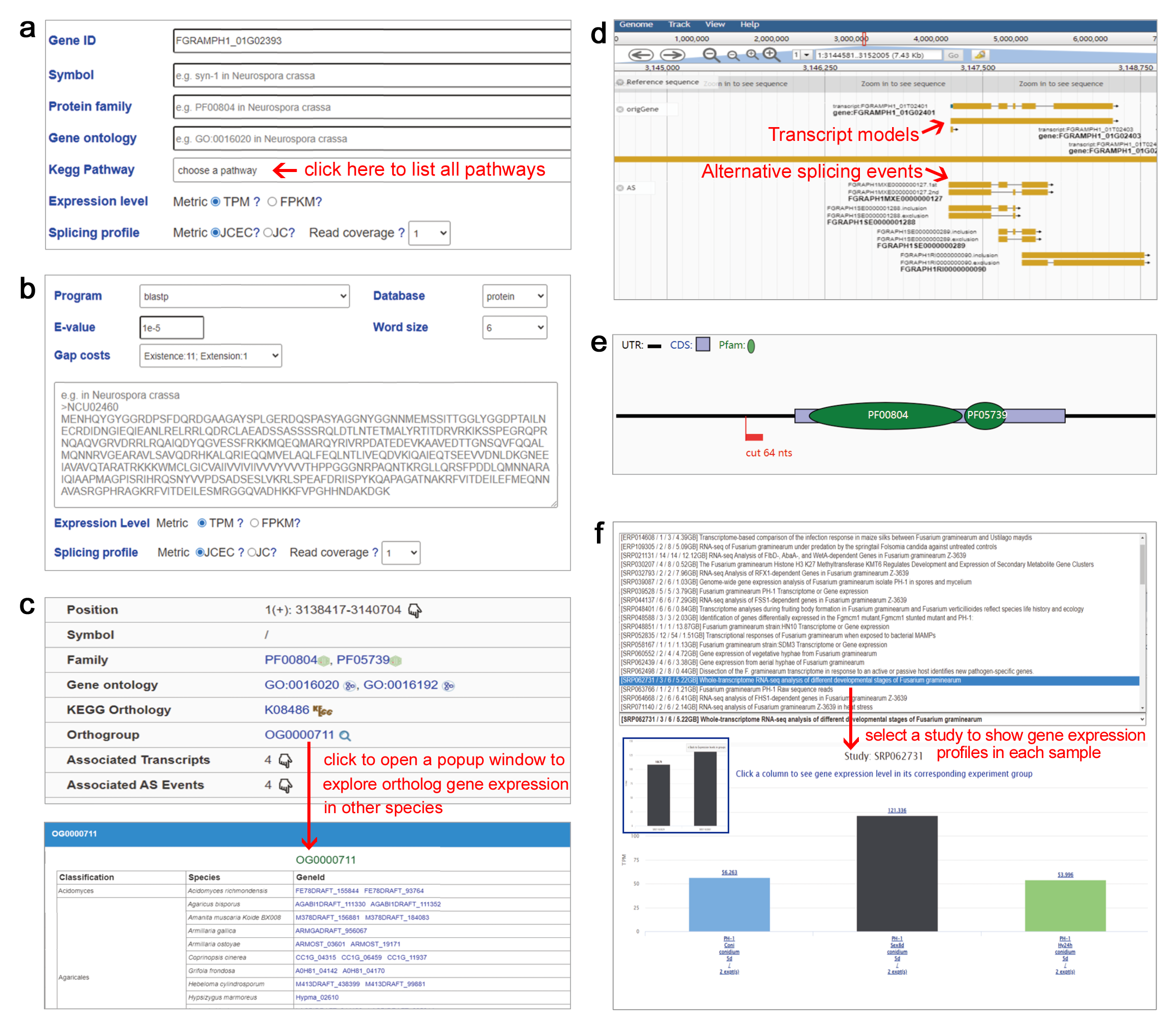


**Supplementary Fig. S1 Querying interfaces and result visualization in FungiExp.**

(a) Genes can be queried by ID, symbol and functional terms in the search page. (b) Genes can be queried by sequence similarity in the blast page. (c) Annotations are listed in the gene top page. Through the ortholog group link, users can open a popup window to explore ortholog gene expression in the other fungal species. (d) JBrowse is embedded to exhibit the structures of gene models and alternative splicing events in genomic context. (e) Illustration for impacts of alternative splicing on a transcript with protein domains. This alternative splicing removes 64 nts in the 3’ UTR region. (f) A list box and a hierarchical bar chart cooperatively show gene expression profiles in a specified study.


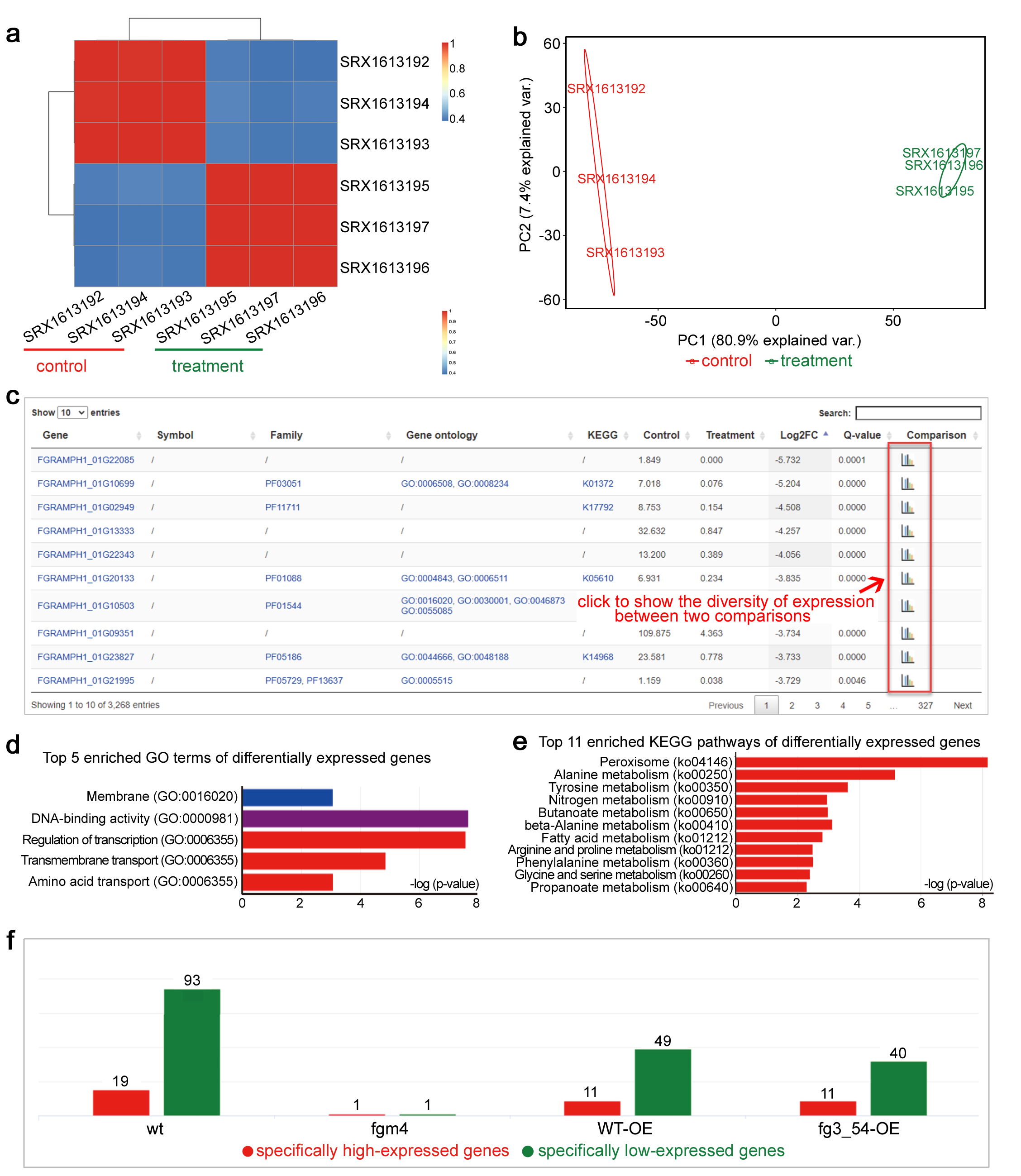


**Supplementary Fig. S2 Visual analysis results and table from differential/specific expression analysis modules.**

(a) The Heatmap plot shows the hierarchical clustering of samples from control and treatment groups. (b) PCA plot of overall gene expression for control and treatment samples. (c) Differentially expressed genes shown in an interactive table. (d) Enriched gene ontology terms for differentially expressed genes. (e) Enriched KEGG pathways for differentially expressed genes. (f) Summary of specifically high- and low-expressed genes in the four sample groups such as wt, fgm4, WT-OE and fg3_54-OE.
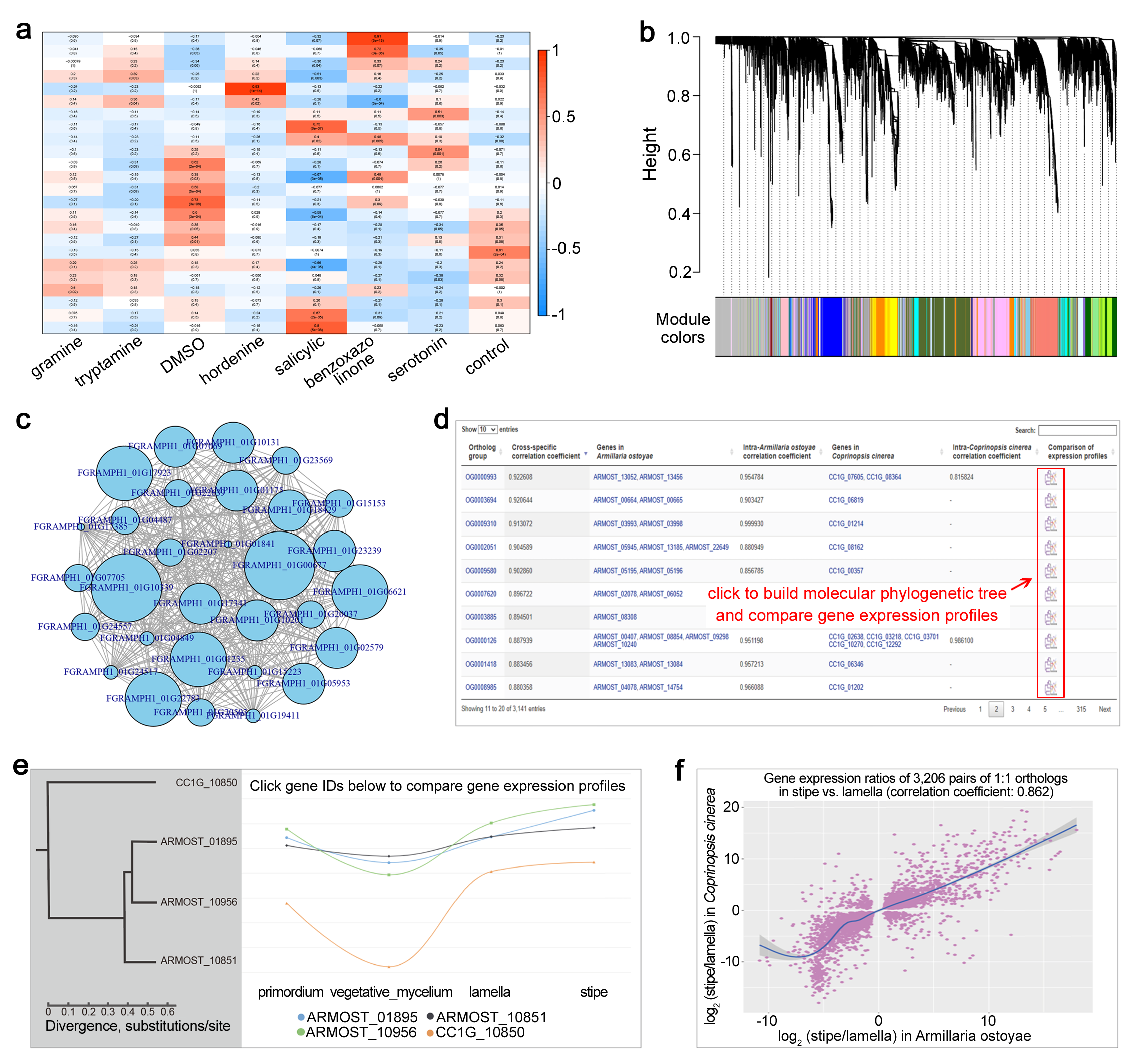


**Supplementary Fig. S3 Visual results of co-expression network and cross-species expression conservation analysis.**

(a) A heatmap shows relationships between gene clusters and sample groups. (b) A gene dendrogram of clustered dissimilarity and module color. (c) A co-expression network corresponding to a gene module. The larger nodes represent the more important hub genes. (d) Ortholog groups listed in an interactive table with correlation coefficients of ortholog gene expression profiles across sample groups. (e) Molecular phylogenetic tree of ortholog genes and their visual expression profiles across four sample groups. (f) Scatter plot of gene expression ratios of 1:1 orthologs (*Coprinopsis cinereal* – *Armillaria ostoyae*) in stipe vs. lamella.


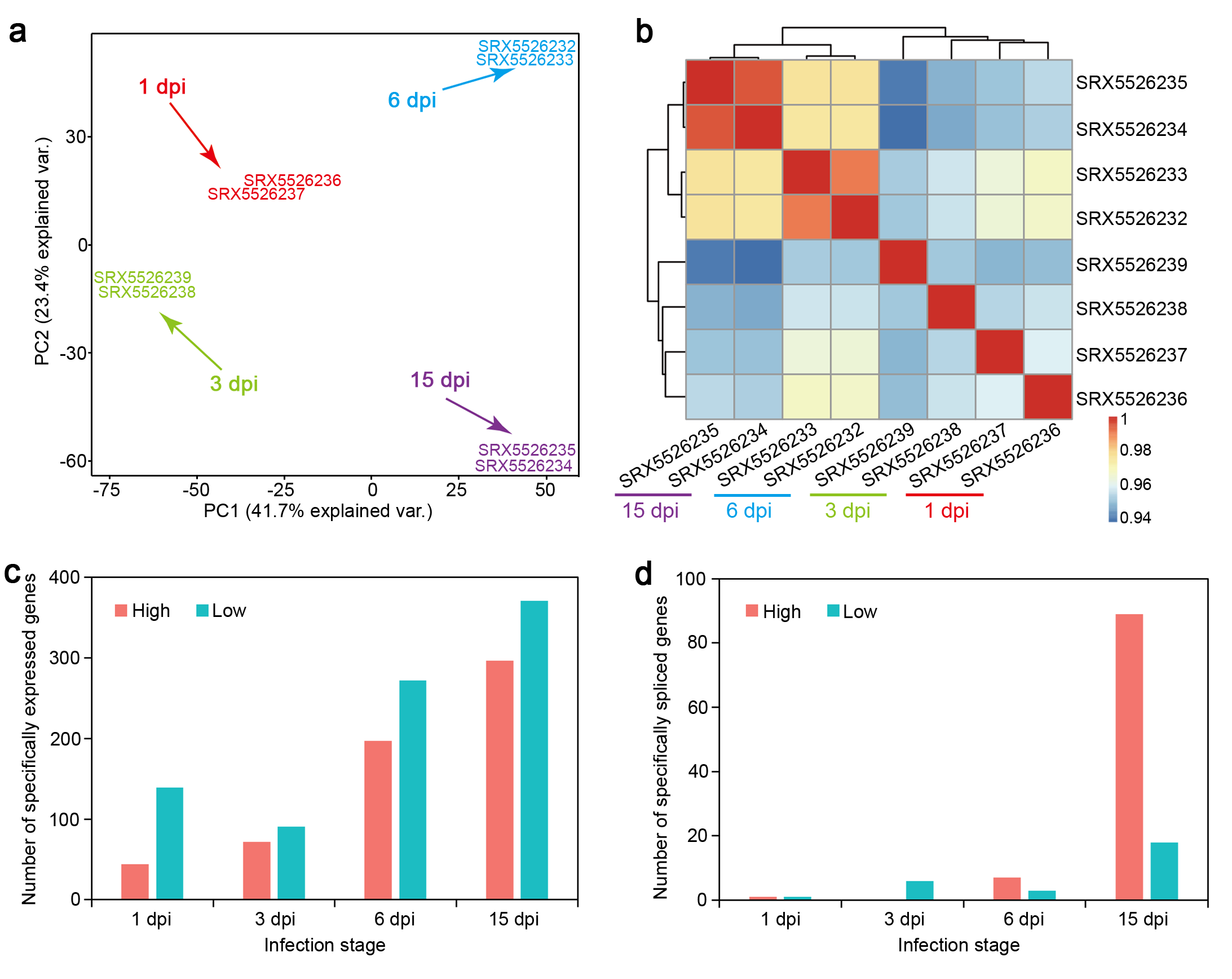


**Supplementary Fig. S4 A case study to explore infection stage-specifically expressed and spliced genes in *U. virens* across four infection stages.**

(a) A PCA plot shows the relationships of overall gene expression profiles of *U. virens* at four infection stages including 1, 3, 6, and 15 dpi. (b) Hierarchical clustering map based on overall alternative splicing PSI values of *U. virens* at four infection stages including 1, 3, 6, and 15 dpi. (c) Number of specifically high- and low-expressed *U. virens* genes at each infection stage. (d) Number of specifically high- and low-spliced *U. virens* genes at each infection stage.
